# Alcohol Consumption Patterns and Chronic Pain: Findings From the 2019 Brazilian National Health Survey

**DOI:** 10.1111/dar.70235

**Published:** 2026-08-18

**Authors:** Mariana Campello de Oliveira, Maria Olivia Pozzolo Pedro, Kae Leopoldo, Douglas Henrique Crispim, João Maurício Castaldelli‐Maia

**Affiliations:** ^1^ Departamento de Psiquiatria Instituto Perdizes, Hospital das Clínicas da Faculdade de Medicina da Universidade de São Paulo São Paulo Brazil; ^2^ Departamento de Psiquiatria Faculdade de Medicina da Universidade de São Paulo São Paulo Brazil; ^3^ Departamento de Psicologia Experimental Instituto de Psicologia da Universidade de São Paulo São Paulo Brazil

**Keywords:** alcohol use, binge drinking, chronic pain, coping mechanisms, low‐ and middle‐income countries

## Abstract

**Introduction:**

Chronic pain and harmful alcohol use are prevalent and disabling conditions with shared neurobiological and social determinants, yet their relationship remains understudied in low‐ and middle‐income countries.

**Methods:**

Cross‐sectional data from 90,846 adults with complete data on the 2019 Brazilian National Health Survey were analysed. Chronic pain was categorised as back pain, rheumatism/arthritis and repetitive strain injury/work‐related musculoskeletal disorder. Alcohol use was assessed by consumption frequency (six categories), binge drinking and average daily intake. Weighted logistic regression models adjusted for sociodemographic and clinical variables were applied.

**Results:**

A U‐shaped association was observed between alcohol consumption frequency and chronic pain: compared with regular‐high frequency drinkers (3–5 days/week, reference), higher odds of chronic pain were observed among near‐daily (odds ratio [OR] 1.73; 95% confidence interval [CI] 1.17–2.56), occasional (OR 1.61; 95% CI 1.20–2.15), infrequent (OR 1.42; 95% CI 1.11–1.82) and regular‐low (1–2 days/week; OR 1.28; 95% CI 1.03–1.60) drinkers. Binge drinking was independently associated with higher odds of chronic pain (OR 1.21; 95% CI 1.03–1.42). Female sex (OR 1.65; 95% CI 1.49–1.83) and depression (OR 2.67; CI 2.32–3.07) showed strong associations with chronic pain. Alcohol quantity (g/day) was not significantly associated with chronic pain in sensitivity analyses.

**Discussion and Conclusions:**

Frequent alcohol use, binge drinking, female sex and depression were independently associated with chronic pain in this low‐ and middle‐income countries population. These associations highlight the importance of addressing behavioural and mental health patterns in pain management. Future longitudinal studies are needed to determine the directionality of these associations.

## 1 Introduction

Chronic pain is a complex and debilitating condition that affects millions of individuals worldwide [1, 2]. Defined as pain persisting for more than three months, it significantly impairs quality of life, limits daily functioning and contributes substantially to years lived with disability [3]. Alcohol use is a leading contributor to the global burden of disease, accounting for over 5% of all deaths and disability‐adjusted life years [4]. Its prolonged use may influence the course and severity of chronic pain through multiple pathways [5].

The co‐occurrence of chronic pain and alcohol use is well‐established and underpinned by a bidirectional relationship. On one hand, individuals may turn to alcohol as a coping mechanism for persistent discomfort, a pattern that can escalate into alcohol use disorder (AUD) [6]. On the other, individuals with AUD are more likely to experience chronic pain compared to the general population [7]. This bidirectional link is supported by shared neurobiological pathways, particularly in brain regions involved in reward processing and emotional regulation. Chronic pain can alter cortical pain processing and contribute to central sensitisation, a state of amplified neural signalling in which the nervous system maintains heightened reactivity to pain stimuli. This sensitisation may lower the threshold for pain perception and increase reliance on alcohol as an analgesic strategy, thereby perpetuating and intensifying the cycle of pain and alcohol use [8].

Beyond neurobiological mechanisms, psychosocial and behavioural factors play an important role in this bidirectional relationship. Anxiety and depression frequently co‐occur with both chronic pain and AUD, and may act as mediating or moderating factors in their interaction [9, 10]. The use of alcohol as a coping strategy for pain‐related distress is particularly relevant, as it may provide short‐term relief while reinforcing maladaptive patterns of consumption over time [11, 12].

The interplay between chronic pain and alcohol use poses particular challenges in low‐ and middle‐income countries (LMIC), where rising rates of non‐communicable conditions coexist with persistent infectious disease burdens within under‐resourced health systems [13, 14]. In parallel, mental health and substance use disorders constitute a major component of the disease burden in LMICs, ranking among the leading causes of disability worldwide [15]. Restricted access to effective pain management and mental health services may increase reliance on alcohol as a self‐medication strategy among individuals with chronic pain [11]. Moreover, socioeconomic vulnerabilities, including poverty and lower educational attainment, are independently associated with both chronic pain and hazardous alcohol use [16], potentially increasing their co‐occurrence in LMICs settings. Cultural norms surrounding alcohol consumption and pain expression, together with the high prevalence of informal labour, may further shape patterns of alcohol use and contribute to work‐related musculoskeletal pain [14, 16].

Consequently, individuals with harmful alcohol use may have undertreated chronic pain, whereas those seeking care for chronic pain may have unrecognised risky alcohol consumption. This bidirectional clinical challenge often remains overlooked because pain management and addiction treatment are typically delivered in separate healthcare settings [15]. Despite the global relevance of these conditions, most existing evidence derives from high‐income countries, limiting its applicability to LMICs contexts.

In this context, the present study aims to examine the association between alcohol use and chronic pain using nationally representative data from the 2019 Brazilian National Health Survey (Pesquisa Nacional de Saúde—PNS) [17], with particular attention to alcohol consumption patterns.

## 2 Methods

This was a cross‐sectional analytical study using secondary data from the PNS 2019, conducted by the Brazilian Institute of Geography and Statistics in partnership with the Ministry of Health. The PNS is a nationally representative household survey designed to assess health conditions, lifestyle behaviours and access to health services among Brazilian adults [18]. The sampling plan was structured in three stages: (i) primary sampling units: census tracts or groups of tracts; (ii) secondary units: households selected within each primary sampling unit; and (iii) tertiary units: one adult (≥ 18 years) was randomly selected within each household to complete the individual questionnaire. The survey included 293,726 total records across all modules. Of these, 90,846 adults with complete data on the individual questionnaire module were eligible for inclusion in the present analyses. The PNS excluded institutionalised individuals, persons living in collective housing and active military personnel, which should be considered when generalising findings to the broader Brazilian population. All analyses incorporated he complex sampling design variables (strata, clusters and sample weights) to produce nationally representative estimates [17, 18].

### 2.1 Measures

#### 2.1.1 Pain

The primary outcome, any chronic pain, was defined as an affirmative response to at least one of three PNS items assessing chronic musculoskeletal conditions. Back pain was assessed by the item: ‘Do you have any chronic back problem, such as chronic back or neck pain, low back pain, sciatica, or problems with the vertebrae or discs?’ (Q084). Rheumatism/arthritis was identified through the item: ‘Has a physician ever given you a diagnosis of arthritis or rheumatism?’(Q079), which encompasses arthritis, rheumatism, osteoarthritis and fibromyalgia, as specified in the PNS instrument. Repetitive strain injury/work‐related musculoskeletal disorder (RSI/WMSD) was assessed by: ‘Has a physician ever given you a diagnosis of RSI/WMSD (repetitive strain injury or work‐related musculoskeletal disorder)?’ (Q088). These three items represent the complete set of chronic pain conditions available in the PNS 2019 individual questionnaire; other conditions such as neuropathic pain and cancer‐related pain are not assessed by the instrument.

#### 2.1.2 Alcohol Consumption

Alcohol use was assessed using multiple self‐reported items from the PNS 2019, allowing the characterisation of different dimensions of alcohol consumption patterns. Consumption frequency was derived from items P027 (‘How often do you usually consume alcoholic beverages?’) and P028a (‘How many days per week do you usually consume alcoholic beverages?’). Six mutually exclusive categories were constructed following the two‐stage structure of the PNS instrument: abstinent, infrequent and occasional were derived from P027, while regular‐low (1–2 days per week), regular‐high (3–5 days per week) and near‐daily (6–7 days per week) were derived from P028a. Regular‐high was designated as the reference category, as it emerged as the consumption pattern associated with the lowest odds of chronic pain in preliminary analyses. In addition, binge drinking (P03201) was defined as the consumption of more than five alcoholic drinks on a single occasion in the past 30 days. This measure was analysed independently to capture episodic high‐intake drinking patterns that may not be reflected by frequency‐based measures alone. Average daily alcohol consumption (g/day of pure ethanol) was additionally derived and categorised as non‐drinker (0 g/day), light (1–12.5 g/day), moderate (12.6–49.9 g/day) and heavy (≥ 50 g/day), following Plens et al. [19].

#### 2.1.3 Demographic Characteristics

The sociodemographic and health‐related variables included were: sex (female/male); age modelled as a continuous variable (in years); race/ethnicity classified according to the Brazilian Institute of Geography and Statistics/Brazilian census categories as White, Brown, Black, Yellow and Indigenous, which are the standard self‐reported categories used in Brazilian national surveys; educational level (primary, secondary, higher); employment status (currently employed vs. not employed); marital status (single, married, divorced, widowed); income (categorised into seven population quintiles: Q1 = lowest, Q7 = highest) and self‐reported depression (yes/no).

#### 2.1.4 Statistical Analysis

All analyses were performed using IBM SPSS Statistics version 30.0 and RStudio Version 2025.9.1, employing the Complex Samples module. Descriptive analyses compared individuals with and without chronic pain across all variables of interest, with weighted proportions and 95% confidence intervals estimated for the Brazilian adult population. Group differences were assessed using Rao–Scott adjusted chi‐square tests, with statistical significance set at *p* < 0.05.

Binary logistic regression models were fitted to evaluate associations between alcohol use and chronic pain outcomes (overall and by subtype). The primary model examined alcohol consumption frequency and binge drinking simultaneously in the full analytic sample. Secondary models examined each pain subtype as a separate outcome, with each model including frequency, binge drinking and each of the other two pain conditions as covariates to account for their overlap. Analyses were restricted to participants with complete data. All models were adjusted for sex, age, education, employment and depression, which showed significant associations with chronic pain after adjustment. Given the a priori hypothesis of a non‐linear association, regular‐high drinkers (3–5 days/week) were designated as the reference category based on preliminary analyses. Multicollinearity was assessed using variance inflation factors (VIF) for all alcohol exposure combinations. Three sensitivity analyses were conducted: (i) alcohol frequency modelled as a continuous variable to test for linear trends; (ii) average daily consumption (g/day) substituted for frequency and binge drinking to assess consistency across exposure operationalisations; and (iii) the primary model re‐estimated using current abstainers as the reference category.

## 3 Results

### 3.1 Sample Characteristics

The sample included 90,846 adults from the PNS, representing a weighted population estimate of approximately 168 million Brazilians. The mean age of the sample was 49.82 years (SE = 0.199; median = 48 years). Participants were aged 18 years or older, consistent with the PNS individual questionnaire eligibility criteria. Sociodemographic factors stratified by chronic pain status are presented in Table 1. Individuals with chronic pain were older, more frequently female and more likely to report depression, unemployment and lower educational attainment (all *p* < 0.001). Although income and marital status were significantly associated with chronic pain in univariate analyses, these associations were not significant after adjustment and were not included in the final models. No significant difference was observed by race/ethnicity (*p* = 0.131).

**TABLE 1:** Sociodemographic and health characteristics by chronic pain status (Brazilian National Health Survey 2019).

Variable	No pain, *n* (%)	Pain, *n* (%)	*p*	OR (95% CI)
Age				
Mean (SE)	47.14 (0.28)	57.73 (0.41)	< 0.001	—
Sex				
Female	33,774 (49.8%)	14,273 (62.3%)	< 0.001	
Male	33,596 (50.2%)	9203 (37.7%)		0.599 (0.564–0.637)
Race/ethnicity				
White	24,293 (42.4%)	8840 (44.4%)	0.131	—
Brown (Parda)	34,373 (44.5%)	11,621 (43.0%)		—
Black	7688 (11.6%)	2657 (10.9%)		—
Yellow	504 (0.9%)	176 (1.1%)		—
Indigenous	504 (0.5%)	180 (0.5%)		—
Marital status				
Single	32,317 (49.4%)	8243 (32.9%)	< 0.001	—
Married	25,475 (39.6%)	9669 (47.3%)		—
Divorced	5054 (5.9%)	2460 (8.9%)		—
Widowed	4524 (5.0%)	3104 (10.9%)		—
Education				
Primary (low)	24,061 (38.1%)	11,073 (51.2%)	< 0.001	—
Secondary (medium)	21,470 (41.9%)	5941 (31.6%)		—
Higher	11,018 (19.9%)	3318 (17.2%)		—
Income				
First (lowest)	7232 (8.5%)	2318 (7.5%)	< 0.001	—
Second	10,805 (15.3%)	3342 (13.4%)		—
Third	19,292 (29.1%)	7114 (29.8%)		
Fourth	16,557 (27.2%)	5909 (28.7%)		—
Fifth	5606 (8.8%)	2006 (9.2%)		—
Sixth	4115 (6.2%)	1439 (6.0%)		—
Seventh (highest)	3743 (4.9%)	1346 (5.4%)		—
Employment				
Employed	37,332 (55.9%)	10,493 (46.8%)	< 0.001	
Unemployed	30,038 (44.1%)	12,983 (53.2%)		0.696 (0.657–0.738)
Depression				
No	62,944 (93.1%)	19,570 (81.3%)	< 0.001	
Yes	4426 (6.9%)	3906 (18.7%)		3.090 (2.837–3.365)
Alcohol consumption frequency				
Abstinent	40,218 (58.1%)	15,212 (61.3%)	< 0.001	—
Infrequent (< 1×/month)	8267 (11.9%)	2826 (12.2%)		—
Occasional (< 1×/week)	2358 (3.5%)	859 (3.9%)		—
Near‐daily (6–7 days/week)	1150 (1.7%)	435 (2.3%)		—
Regular‐low (1–2 days/week)	12,702 (20.2%)	3420 (16.5%)		—
Regular‐high (3–5 days/week)	2675 (4.5%)	724 (3.8%)		—
Binge drinking (≥ 5 doses/occasion in last 30 days)				
Yes	10,097 (15.3%)	2672 (12.2%)	< 0.001	
No	57,273 (84.7%)	20,804 (87.8%)		1.303 (1.192–1.423)
Alcohol quantity (g/day)				
Non‐drinker (0 g/day)	50,843 (73.5%)	18,897 (77.4%)	< 0.001	—
Light (1–12.5 g/day)	8542 (14.5%)	2584 (13.6%)		—
Moderate (12.6–49.9 g/day)	6628 (10.1%)	1618 (7.3%)		—
Heavy (≥ 50 g/day)	1357 (1.9%)	377 (1.7%)		—
Total	67,370	23,476		

*Note:* *n* = unweighted sample size; (%) = weighted population estimates. *N* = 90,846 (weighted population estimate: ~168 million). Bivariate ORs shown for reference; adjusted ORs reported in Tables 2, 3, 4, 5.

Abbreviations: CI, confidence interval; OR, odds ratio; SE, standard error.

### 3.2 Multivariate Models: Any Chronic Pain

Alcohol consumption frequency, binge drinking and quantity all differed significantly between groups in bivariate analyses (Table 1). In the adjusted model for any chronic pain (Table 2), alcohol frequency showed a significant overall association (*p* = 0.001), with a U‐shaped pattern relative to the regular‐high reference group (3–5 days/week): near‐daily, occasional, infrequent and regular‐low drinkers all showed significantly higher odds of chronic pain, while abstinent individuals showed a similar but non‐significant trend. Binge drinking was independently associated with higher odds of chronic pain (odds ratio [OR] 1.21, *p* = 0.018). Female sex, depression and age were the strongest independent predictors (Table 2). The sex × alcohol frequency interaction term was not statistically significant (Wald *F* = 0.413, *p* = 0.840), indicating that the U‐shaped association did not differ significantly by sex.

**TABLE 2:** Factors associated with any chronic pain.

Variable	Adjusted OR	95% CI	*p*
Alcohol frequency (ref = regular‐high, 3–5 days/week)			0.001
Abstinent vs. regular‐high	1.171	0.920–1.489	0.199
Infrequent (< 1×/months) vs. regular‐high	1.421	1.108–1.823	0.006
Occasional (< 1×/weeks) vs. regular‐high	1.605	1.200–2.147	0.001
Near‐daily (6–7 days/week) vs. regular‐high	1.729	1.170–2.557	0.006
Regular‐low (1–2 days/week) vs. regular‐high	1.283	1.027–1.604	0.028
Binge drinking			
Yes vs. no	1.212	1.034–1.420	0.018
Sex			
Female vs. male	1.654	1.494–1.831	< 0.001
Depression			
Yes vs. no	2.669	2.319–3.072	< 0.001
Age (per year)			
1‐year increment	1.035	1.031–1.038	< 0.001
Education			0.038
Low vs. high	1.196	0.903–1.583	0.211
Medium vs. high	1.061	0.796–1.416	0.685
Employment			
Unemployed vs. employed	0.991	0.900–1.090	0.846

*Note:* Nagelkerke *R* ^2^ = 0.108. Variance inflation factors: frequency = 1.760, binge = 1.749, max = 1.760. *N* = 90,846 (weighted population estimate: ~168 million).

Abbreviations: CI, confidence interval; OR, odds ratio.

As sensitivity analyses, alcohol frequency modelled continuously (days per week) showed no significant linear association with chronic pain (Wald *F* = 0.462, *p* = 0.497), consistent with the U‐shaped pattern observed in categorical analyses. Alcohol quantity (g/day) was not significantly associated with chronic pain in any specification, either categorically or continuously (*p* > 0.05 across all models, Table S1). When quantity was modelled jointly with frequency and binge drinking, VIF exceeded conventional thresholds (frequency VIF > 4, quantity VIF > 5), whereas frequency and binge drinking alone showed acceptable collinearity (VIF < 2.0 for both); the latter were therefore retained as the primary alcohol exposure variables.

### 3.3 Overlap Among Pain Subtypes

Substantial overlap was observed among the three musculoskeletal pain conditions (Figure S1). Back pain was the most prevalent condition (*n* = 19,206; 21.1% of the analytic sample), and coexistence of all three conditions was observed in 292 individuals (1.2% of those with any pain). Given this overlap, each subtype model included the other two conditions as covariates.

### 3.4 Multivariate Models: Pain Subtypes

For back pain (Table 3), alcohol frequency showed a significant overall association (*p* = 0.003), with a U‐shaped pattern similar to any chronic pain: near‐daily (OR 1.85), occasional (OR 1.53) and regular‐low (OR 1.29) drinkers all showed significantly higher odds than regular‐high drinkers. Binge drinking was independently associated with back pain (OR 1.23, *p* = 0.020). Back pain was strongly associated with rheumatism/arthritis (OR 3.08) and RSI/WMSD (OR 2.89).

**TABLE 3:** Factors associated with back pain (*N* = 90,846).

Variable	Adjusted OR	95% CI	*p*
Alcohol frequency (ref = regular‐high, 3–5 days/week)			0.003
Abstinent vs. regular‐high	1.142	0.871–1.499	0.336
Infrequent vs. regular‐high	1.300	0.996–1.696	0.054
Occasional vs. regular‐high	1.529	1.101–2.122	0.011
Near‐daily vs. regular‐high	1.850	1.213–2.821	0.004
Regular‐low vs. regular‐high	1.290	1.005–1.655	0.045
Binge drinking			
Yes vs. no	1.230	1.034–1.465	0.020
Sex			
Female vs. male	1.343	1.207–1.495	< 0.001
Depression			
Yes vs. no	2.371	2.031–2.769	< 0.001
Age (per year)			
1‐year increment	1.022	1.018–1.026	< 0.001
Education			0.183
Low vs. high	1.030	0.760–1.397	0.848
Medium vs. high	0.930	0.678–1.275	0.650
Employment			
Unemployed vs. employed	1.030	0.929–1.143	0.569
Comorbid pain conditions			
RSI/WMSD: yes vs. no	2.887	2.291–3.643	< 0.001
Rheumatism/arthritis: yes vs. no	3.077	2.632–3.597	< 0.001

*Note:* Variance inflation factors: freq = 1.759, binge = 1.740, rheumatism = 1.063, repetitive strain injury/work‐related musculoskeletal disorder (RSI/WMSD) = 1.014, max = 1.759. *N* = 90,846 (weighted population estimate: ~168 million).

Abbreviations: CI, confidence interval; OR, odds ratio.

For rheumatism/arthritis (Table 4), alcohol frequency also showed a significant overall association (*p* = 0.006), but with a distinct pattern: infrequent (OR 2.09) and abstinent (OR 1.76) drinkers showed the highest odds relative to regular‐high drinkers, while near‐daily drinkers showed no difference (OR 0.99, *p* = 0.980). Binge drinking was not significantly associated with rheumatism/arthritis (*p* = 0.368). Rheumatism/arthritis was strongly associated with back pain (OR 3.22) and RSI/WMSD (OR 1.82).

**TABLE 4:** Factors associated with rheumatism/arthritis.

Variables	Adjusted OR	95% CI	*p*
Alcohol frequency (ref = regular‐high, 3–5 days/week)			0.006
Abstinent vs. regular‐high	1.755	1.216–2.533	0.003
Infrequent vs. regular‐high	2.091	1.380–3.169	< 0.001
Occasional vs. regular‐high	1.477	0.906–2.408	0.118
Near‐daily vs. regular‐high	0.990	0.450–2.176	0.980
Regular‐low vs. regular‐high	1.487	1.039–2.129	0.030
Binge drinking			
Yes vs. no	1.154	0.845–1.576	0.368
Sex			
Female vs. male	2.346	1.936–2.842	< 0.001
Depression			
Yes vs. no	2.024	1.647–2.488	< 0.001
Age (per year)			
1‐year increment	1.063	1.054–1.071	< 0.001
Education			0.019
Low vs. high	1.736	1.095–2.752	0.019
Medium vs. high	1.475	0.922–2.358	0.105
Employment			
Unemployed vs. employed	1.069	0.903–1.265	0.440
Comorbid pain conditions			
Back pain: yes vs. no	3.216	2.748–3.765	< 0.001
RSI/WMSD: yes vs. no	2.045	1.488–2.810	< 0.001

*Note:* Variance inflation factors: freq = 1.760, binge = 1.750, back pain = 1.053, repetitive strain injury/work‐related musculoskeletal disorder (RSI/WMSD) = 1.022, max = 1.760. *N* = 90,846 (weighted population estimate: ~168 million).

Abbreviations: CI, confidence interval; OR, odds ratio.

For RSI/WMSD (Table 5), alcohol frequency (overall *p* = 0.418) and binge drinking (*p* = 0.471) were not significantly associated with RSI/WMSD. Unlike the other subtypes, unemployment was associated with lower odds of RSI/WMSD (OR 0.604, *p* < 0.001). RSI/WMSD was strongly associated with back pain (OR 2.92) and rheumatism/arthritis (OR 2.05).

**TABLE 5:** Factors associated with RSI/WMSD.

Variables	Adjusted OR	95% CI	*p*
Alcohol frequency (ref = regular‐high, 3–5 days/week)			0.418
Abstinent vs. regular‐high	0.788	0.441–1.407	0.420
Infrequent vs. regular‐high	1.083	0.571–2.056	0.807
Occasional vs. regular‐high	0.982	0.490–1.970	0.960
Near‐daily vs. regular‐high	1.295	0.568–2.948	0.539
Regular‐low vs. regular‐high	0.919	0.543–1.553	0.751
Binge drinking			
Yes vs. no	0.858	0.565–1.302	0.471
Sex			
Female vs. male	1.906	1.451–2.503	< 0.001
Depression			
Yes vs. no	1.833	1.410–2.381	< 0.001
Age (per year)			
1‐year increment	1.020	1.013–1.027	< 0.001
Education			0.236
Low vs. high	1.658	0.917–2.998	0.094
Medium vs. high	1.511	0.842–2.710	0.166
Employment			
Unemployed vs. employed	0.604	0.477–0.764	< 0.001
Comorbid pain conditions			
Rheumatism/arthritis: yes vs. no	1.822	1.308–2.539	< 0.001
Back pain: yes vs. no	2.920	2.307–3.696	< 0.001

*Note:* Variance inflation factors: freq = 1.760, binge = 1.750, rheumatism = 1.104, back pain = 1.065, max = 1.760. *N* = 90,846 (weighted population estimate: ~168 million).

Abbreviations: CI, confidence interval; OR, odds ratio.

## 4 Discussion

In this large, nationally representative Brazilian sample, alcohol consumption frequency showed a U‐shaped association with chronic pain: compared to regular‐high drinkers (3–5 days/week), higher odds of chronic pain were observed across all other frequency categories. In addition, binge drinking was independently associated with chronic pain after full adjustment (OR 1.21), while alcohol quantity (g/day) was not significantly associated with chronic pain in any specification examined. This study contributes new evidence on these interactions from LMICs, where such data are scarce [15]. It is crucial to emphasise that the cross‐sectional nature of this analysis precludes any determination of causality. Therefore, the following discussion will explore several plausible, and at times competing, hypotheses to explain these correlational results. Although frequency and binge drinking were modelled simultaneously, their independent associations suggest distinct pathways linking alcohol patterns to chronic pain risk.

The U‐shaped pattern observed across frequency categories is broadly consistent with Zale et al. [12], who described a curvilinear relationship between alcohol use and pain outcomes, with regular use associated with better outcomes relative to both abstinence and excessive consumption. Notably, alcohol quantity was not significantly associated with chronic pain in sensitivity analyses, suggesting that consumption patterns beyond quantity alone warrant attention in this population [12].

Several non‐mutually exclusive hypotheses may explain the observed U‐shaped pattern. Among infrequent and abstinent individuals, higher odds of chronic pain may reflect a pattern in which pain limits social functioning and participation in activities where alcohol is typically consumed [20]. Among near‐daily drinkers, alcohol may be used as an accessible but maladaptive coping strategy for symptom relief, while simultaneously contributing to pain through systemic inflammation, sleep disruption or neuroadaptive changes [8, 11]. Notably, although alcohol consumers reported lower pain intensity, they paradoxically experienced greater pain interference in daily activities [5, 12], reinforcing the maladaptive nature of alcohol as a coping strategy across frequency patterns.

Consistent with existing literature, chronic pain was strongly associated with female sex, older age and depression [6, 10, 21]. The co‐occurrence with depression is particularly notable, as both conditions engage overlapping neurobiological pathways and may mutually reinforce one another [8, 9]. This highlights a vulnerable subgroup for whom integrated treatment approaches are essential.

Interestingly, the association between frequent alcohol use and pain varied by subtype. For back pain, a U‐shaped pattern similar to any chronic pain was observed, with near‐daily drinkers showing the highest odds (OR 1.85). For rheumatism/arthritis, the pattern was distinct: infrequent and abstinent individuals showed the highest odds (OR 2.09 and OR 1.76, respectively), while near‐daily drinkers showed no significant difference from regular‐high drinkers, suggesting a J‐shaped rather than U‐shaped pattern. This suggests that the underlying pathophysiology of the pain condition may moderate its relationship with alcohol. Pain with a stronger inflammatory component, such as back pain or rheumatic conditions, may be perceived as more responsive to alcohol's short‐term analgesic effects [22, 23], whereas pain from mechanical or work‐related strain (RSI/WMSD) may be less influenced by this pattern of consumption. In rheumatic and musculoskeletal conditions, alcohol and smoking have been associated with worse clinical outcomes [24].

Given that data were collected in 2019, prior to the COVID‐19 pandemic, the generalisability of findings to current contexts warrants consideration. The pandemic has been associated with significant increases in both alcohol consumption and chronic pain prevalence globally [25, 26], suggesting that the associations observed here may underestimate the current burden of these co‐occurring conditions in Brazil and other LMICs. Data from the next wave of the PNS will provide an important opportunity to examine whether the U‐shaped pattern observed here is robust across different epidemiological contexts. From a public health perspective, these findings underscore the need for integrated care in LMICs, where access to specialised treatment for pain, mental health and substance use disorder is often fragmented and limited [15, 27]. The bidirectional relationship between chronic pain and alcohol use, where persistent pain may increase alcohol use and alcohol use may exacerbate pain through neuroadaptive changes [12, 28], highlights the importance of routine screening for alcohol patterns and depression in primary care patients presenting with chronic pain. In resource‐limited contexts, task‐sharing strategies and evidence‐based psychosocial interventions should be prioritised to reduce the cumulative burden of these co‐occurring conditions [29].

### 4.1 Limitations

Several limitations should be acknowledged. The cross‐sectional design precludes causal inference, preventing determination of whether alcohol use precedes chronic pain or vice versa. Alcohol consumption was assessed using self‐reported measures, without validated screening instruments such as the Alcohol Use Disorders Identification Test, limiting the ability to identify AUD or alcohol‐related consequences beyond consumption patterns. The absence of past drinking history prevented empirical testing of the ‘sick quitter’ hypothesis [30, 31]. Important confounding variables were not available, including analgesic use, other substance use disorders (e.g., tobacco and cannabis) and specific pain treatments, which may have influenced the observed associations. The three pain subtypes examined represent the complete set available in the PNS 2019 instrument; other conditions such as neuropathic and cancer‐related pain could not be assessed. Finally, as data were collected in 2019, findings may have limited generalisability to current contexts given documented post‐pandemic changes in both alcohol use and chronic pain burden, and the next wave of the PNS will provide an important opportunity to examine whether the associations observed here have shifted in the post‐pandemic period. Despite these limitations, this study provides robust population‐based evidence on the association between alcohol consumption patterns and chronic pain in a large, nationally representative Brazilian sample, with important implications for understanding this relationship in LMICs.

## 5 Conclusion

In this nationally representative study, alcohol consumption frequency showed a U‐shaped association with chronic pain, with both infrequent/abstinent and near‐daily drinking patterns associated with higher odds of chronic pain relative to regular‐high frequency drinkers. Binge drinking was independently associated with chronic pain after full adjustment. The association varied by pain subtype, with frequency and binge drinking significantly associated with back pain, a distinct pattern observed for rheumatism/arthritis, and no significant alcohol associations found for RSI/WMSD. The frequent co‐occurrence of chronic pain with depression and female sex further highlights the complex biopsychosocial context underlying these associations and the importance of integrated screening approaches. These findings reinforce the need for integrated care approaches that incorporate mental health and substance use assessment into chronic pain management. In LMICs, where access to multidisciplinary care is limited, targeted public health strategies are needed to address these conditions concurrently and reduce inequities in care.

## Author Contributions

**Mariana Campello de Oliveira:** conceptualisation, methodology, writing – original draft preparation and writing – review and editing. **João Maurício Castaldelli‐Maia:** conceptualisation, methodology, formal analysis, writing – review and editing and supervision. **Maria Olivia Pozzolo Pedro:** formal analysis, writing – review and editing and visualisation. **Kae Leopoldo:** writing – review and editing and visualisation. **Douglas Henrique Crispim:** visualisation. All authors have read and agreed to the published version of the manuscript.

## Funding

The authors have nothing to report.

## Conflicts of Interest

The authors declare no conflicts of interest.

## Supporting information


**Figure S1:** Venn diagram illustrating the overlap of back pain, rheumatism/arthritis and RSI/WMSD among adults in the PNS 2019.

**Table S1:** Sensitivity analyses: Alcohol exposure modelled as (A) frequency in days/week as a continuous variable (OLS regression) and (B) average daily quantity in g/day (complex samples logistic regression).
**Table S2:** Sensitivity analysis: Adjusted odds ratios for chronic pain (any) using current abstainers as the reference category.

## Data Availability

The data that support the findings of this study are openly available in 2019—Pesquisa Nacional de Saúde (PNS) at https://www.pns.icict.fiocruz.br/wp‐content/uploads/2021/12/liv101846.pdf, reference number ISBN 978‐65‐87201‐76‐4.
