# Supplementary material for: Alcohol Consumption Patterns and Chronic Pain: Findings From the 2019 Brazilian National Health Survey
Source: Drug Alcohol Rev. 2026 Aug 18;45(6):e70235. doi: 10.1111/dar.70235 (PMC13487351; doi:10.1111/dar.70235)
Supplement: Supplementary file 1 [file DAR-45-0-s001.docx]

Supplementary Figure 1 - Venn diagram illustrating the overlap of back pain, rheumatism/arthritis, and RSI/WMSD among adults in the PNS 2019. Values correspond to the number of individuals within each intersection.
