# Supplementary material for: Alcohol Consumption Patterns and Chronic Pain: Findings From the 2019 Brazilian National Health Survey
Source: Drug Alcohol Rev. 2026 Aug 18;45(6):e70235. doi: 10.1111/dar.70235 (PMC13487351; doi:10.1111/dar.70235)
Supplement: Supplementary file 2 [file DAR-45-0-s002.docx]

**Supplementary Table 1. Sensitivity analyses: alcohol exposure modeled as (A) frequency in days/week as a continuous variable (OLS regression) and (B) average daily quantity in g/day (Complex Samples logistic regression).**

| **Variable** | **B** | **SE** | **Beta (std.)** | **p-value** |
| --- | --- | --- | --- | --- |
| **Panel A- Alcohol frequency as continuous variable (days/week)** | | | | |
| Alcohol frequency (days/week) | 0.000 | 0.000 | .014 | .108 |
| Depression | 0.182 | 0.013 | .123 | <.001 |
| Female sex | -0.071 | 0.007 | -.086 | <.001 |
| Education | -0.015 | 0.007 | -.021 | .020 |
| Employment | -0.006 | 0.008 | -.007 | .455 |
| Age | 0.005 | 0.000 | .177 | <.001 |
| **R² (model)** | 0.057 |  |  |  |
| **N (unweighted)** | 12,903 |  |  |  |

*Panel A: OLS regression, unweighted (N = 12,903). Dependent variable: chronic pain (binary). Alcohol frequency as continuous variable (days/week) was not significantly associated with chronic pain (B = 0.000, p = .108), confirming the non-monotonic pattern observed in the primary categorical model.*

| **Variable** | **OR** | **95% CI Lower** | **95% CI Upper** | **p-value** |
| --- | --- | --- | --- | --- |
| **Panel B - Alcohol quantity (g/day), Complex Samples Logistic Regression** | | | | |
| Light (1–12.5 g/day) vs. Non-drinker | — | — | — | .130ᵃ |
| Moderate (12.6–49.9 g/day) | — | — | — |  |
| Heavy (≥50 g/day) | — | — | — |  |
| Depression | 2.671 | 2.323 | 3.072 | <.001 |
| Female sex | 1.616 | 1.462 | 1.787 | <.001 |
| **Nagelkerke R²** | 0.105 |  |  |  |
| **N (unweighted)** | 38,704 |  |  |  |

*Panel B: alcohol_NLMH overall effect: Wald F(3, 2869) = 1.884, p = .130 (non-significant). Individual OR estimates for alcohol quantity categories are not reported due to overall non-significance.*

**Supplementary Table 2. Sensitivity analysis: adjusted odds ratios for chronic pain (any) using current abstainers as the reference category.**

| **Variable** | **OR** | **95% CI Lower** | **95% CI Upper** | **p-value** |
| --- | --- | --- | --- | --- |
| **Alcohol consumption frequency (ref = Abstinent)** | | | | |
| Infrequent (<1×/month) | 1.214 | 1.062 | 1.387 | .018 |
| Occasional (<1×/week) | 1.371 | 1.108 | 1.697 | .006 |
| Near-daily (6–7 d/wk) | 1.477 | 1.058 | 2.062 | .022 |
| Regular-low (1–2 d/wk) | 1.096 | 0.950 | 1.264 | .208 |
| Regular-high (3–5 d/wk) | 0.854 | 0.671 | 1.086 | .197 |
| **Binge drinking** | | | | |
| No vs. Yes (ref) | 0.825 | 0.704 | 0.967 | .018 |
| **Covariates** | | | | |
| Female sex | 1.654 | 1.494 | 1.831 | <.001 |
| Depression (yes vs. no) | 2.669 | 2.319 | 3.072 | <.001 |
| Education | — | — | — | .038 |
| Employment (unemployed vs. employed) | 0.991 | 0.900 | 1.090 | .846 |
| Age (continuous) | — | — | — | <.001 |
| **Nagelkerke R²** | 0.108 |  |  |  |
| **Unweighted N** | 38,704 |  |  |  |

*OR = Odds Ratio; CI = Confidence Interval.*
